# Higher Serum Direct Bilirubin Levels Were Associated with a Lower Risk of Incident Chronic Kidney Disease in Middle Aged Korean Men

**DOI:** 10.1371/journal.pone.0075178

**Published:** 2014-02-20

**Authors:** Seungho Ryu, Yoosoo Chang, Yiyi Zhang, Hee-Yeon Woo, Min-Jung Kwon, Hyosoon Park, Kyu-Beck Lee, Hee Jung Son, Juhee Cho, Eliseo Guallar

**Affiliations:** 1 Department of Occupational and Environmental Medicine, Kangbuk Samsung Hospital, Sungkyunkwan University, School of Medicine, Seoul, South Korea; 2 Department of Epidemiology, Johns Hopkins Bloomberg School of Public Health, Baltimore, Maryland, United States of America; 3 Center for Cohort Studies, Total Healthcare Center, Kangbuk Samsung Hospital, Sungkyunkwan University, School of Medicine, Seoul, South Korea; 4 Department of Medicine and Welch Center for Prevention, Epidemiology, and Clinical Research, Johns Hopkins Medical Institutions, Baltimore, Maryland, United States of America; 5 Department of Laboratory Medicine, Kangbuk Samsung Hospital, Sungkyunkwan University, School of Medicine, Seoul, South Korea; 6 Department of Internal Medicine, Kangbuk Samsung Hospital, Sungkyunkwan University, School of Medicine, Seoul, South Korea; 7 Department of Internal Medicine, Samsung Medical Center, Gangnam-Gu, Seoul, Korea; 8 Samsung Comprehensive Cancer Center, Samsung Medical Center, Gangnam-Gu, Seoul, Seoul, Korea; University of Louisville, United States of America

## Abstract

**Background:**

The association between serum bilirubin levels and incident chronic kidney disease (CKD) in the general population is unknown. We aimed to examine the association between serum bilirubin concentration (total, direct, and indirect) and the risk of incident CKD.

**Methods and Findings:**

Longitudinal cohort study of 12,823 Korean male workers 30 to 59 years old without CKD or proteinuria at baseline participating in medical health checkup program in a large worksite. Study participants were followed for incident CKD from 2002 through 2011. Estimated glomerular filtration rate (eGFR) was estimated by using the CKD-EPI equation. CKD was defined as eGFR <60 mL/min per 1.73 m^2^. Parametric Cox models and pooled logistic regression models were used to estimate adjusted hazard ratios for incident CKD. We observed 238 incident cases of CKD during 70,515.8 person-years of follow-up. In age-adjusted models, the hazard ratios for CKD comparing quartiles 2–4 vs. quartile 1 of serum direct bilirubin were 0.93 (95% CI 0.67–1.28), 0.88 (0.60–1.27) and 0.60 (0.42–0.88), respectively. In multivariable models, the adjusted hazard ratio for CKD comparing the highest to the lowest quartile of serum direct bilirubin levels was 0.60 (95% CI 0.41–0.87; P trend = 0.01). Neither serum total nor indirect bilirubin levels were significantly associated with the incidence of CKD.

**Conclusions:**

Higher serum direct bilirubin levels were significantly associated with a lower risk of developing CKD, even adjusting for a variety of cardiometabolic parameters. Further research is needed to elucidate the mechanisms underlying this association and to establish the role of serum direct bilirubin as a marker for CKD risk.

## Introduction

Chronic kidney disease (CKD) is an increasingly common public health problem [1], [2]. In the US, the prevalence of CKD increased from 10.0% in 1988–1994 to 13.1% in 1999–2004 [1], and similar increases are being observed worldwide. Persons with CKD have high rates of morbidity, mortality, health-care utilization, and progression to end-stage renal disease, with its corresponding complications [3], [4]. While some determinants of CKD, including hypertension and diabetes, are well established, the natural history of the disease is incompletely understood and there is substantial interest in the identification of novel risk factors for CKD.

Bilirubin is a product of heme catabolism that may have potent antioxidant and cytoprotective properties [5], [6]. In experimental studies, serum bilirubin plays a significant role in renal ischemia-reperfusion injury [7], [8] and, in patients with IgA nephropathy, mildly elevated serum bilirubin levels were negatively associated with the incidence of end-stage renal disease [9]. Furthermore, cross-sectional [10]–[14] and prospective [15]–[18] studies have reported negative associations between bilirubin concentrations and coronary artery disease, peripheral vascular disease, carotid intimal-medial thickness, stroke, non-alcoholic fatty liver and metabolic syndrome [19], disorders that share common etiologic mechanisms with CKD.

The association between serum bilirubin concentrations and CKD is controversial. A number of cross-sectional studies have shown a positive association between serum total bilirubin concentration and estimated glomerular filtration rate (eGFR) [20]–[22]. A retrospective study showed that higher serum bilirubin concentrations were associated with lower risk of contrast-induced nephropathy and fewer cardiovascular events in the patients with angina pectoris or acute myocardial infarction [23]. Two studies, however, found that higher bilirubin concentrations were significantly associated with lower eGFR both in hospital-based unselected outpatients and in the US general population [24], [25]. No longitudinal study has evaluated the prospective association between serum bilirubin levels and incident CKD in the general population. The goal of this study was thus to evaluate the prospective association between bilirubin concentration (total, direct, and indirect) and the risk for CKD. We also assessed if this association persisted when time-dependent changes in serum bilirubin levels and in other potential confounders were taken into account.

## Methods

### Study Population

The study population was comprised of male workers from one of the largest semiconductor manufacturing companies in Korea and its 13 affiliates [26], [27]. In Korea, the Industrial Safety and Health Law requires employees to participate in annual or biennial health examinations. The present cohort included all male workers 30–59 years of age from the above mentioned semiconductor companies who participated in a comprehensive health examination at the Kangbuk Samsung Hospital in Seoul, Korea in 2002 (N = 15,347). We excluded 1,045 men who had one or more of the following exclusion criteria: history of kidney disease (N = 38); dipstick-positive proteinuria (N = 260); glomerular filtration rate (GFR) of <60 mL/min/1.73 m^2^ at baseline (N = 261); or missing data on their past medical history, serum bilirubin, or urinalysis (N = 659), resulting in 14,302 eligible subjects at the baseline (2002) examination. The final sample size consisted of 12,823 elegible participants who were reexamined at the same hospital, annually or biennially, over a period of 7 years until December 2011.

This study was approved by the Institutional Review Board of the Kangbuk Samsung Hospital. The informed consent requirement for this study was exempted by the Institutional Review Board because researchers only accessed retrospectively a de-identified database for analysis purposes.

### Measurements

Baseline and follow-up examinations were conducted at the Kangbuk Samsung Hospital Health Screening Center. Study participants were examined annually or biennially until December 2011. Health examinations collected data on medical history, medication use, health-related behaviors, physical measurements, and serum biochemical measurements [26]. Questions pertaining to alcohol intake included weekly frequency of alcohol consumption and usual daily amount of consumption. Questionnaire data were also used to identify current smokers and to assess the weekly frequency of moderate- or vigorous-intensity physical activity. Body weight was measured with light clothing and without shoes to the nearest 0.1 kilogram using a digital scale. Height was measured to the nearest 0.1 centimeter. Body mass index (BMI) was calculated as weight in kilograms divided by height in meters squared. Trained nurses measured sitting blood pressure with a standard mercury sphygmomanometer.

Blood specimens were sampled from the antecubital vein after more than 12 hours of fasting. Serum levels of glucose, uric acid, total cholesterol, triglyceride (TG), low-density lipoprotein (LDL) cholesterol, high-density lipoprotein (HDL) cholesterol, gamma-glutamyltransferase (GGT), alanin aminotransferase (ALT), aspartate aminotransferase (AST), total bilirubin and direct bilirubin were measured using Bayer Reagent Packs (Bayer Diagnostics, Leverkusen, Germany) on an automated chemistry analyzer (Advia 1650™ Autoanalyzer; Bayer Diagnostics, Leverkusen, Germany).

Serum total and direct bilirubin were measured with the vanadate oxidation method. Regular calibration and quality control measurements were performed throughout the study period using a validated calibrator and quality control materials. The within-run and total coefficients of variation for total bilirubin determinations were <2.1% for low level quality control specimens and <1.6% for high level quality control specimens. For direct bilirubin, the total and within run coefficients of variation were <5.9% for low level and <3.3% for high level quality control specimens. Serum creatinine was measured with the kinetic alkaline picrate (Jaffe) method. The within-run and total coefficients of variation for creatinine determinations were <3.0% for low level and <1.5% for high level quality control specimens for the duration of the study. We estimated eGFR using the CKD Epidemiology Collaboration (CKD-EPI) equation [28] as follows: GFR (mL/min/1.73 m^2^ body surface area) = 141×min(serum creatinine/0.9,1)^–0.411^×max(serum creatinine/0.9,1)^−1.209^×0.993^age^. CKD was defined as an eGFR <60 mL/min/1.73 m^2^.

All the subjects in this study had a urinalysis at the time of the baseline examination. Urine protein was determined at each examination by single urine dipstick semi-quantitative analysis (URiSCAN® Urine strip; YD Diagnostics, Yong-In, Korea). Dipstick urinalysis was performed on fresh, midstream urine samples collected in the morning. The amount of urine protein was reported as the following six grades: absent, trace, 1+, 2+, 3+ and 4+, corresponding approximately to undetectable, 10, 30, 100, 300, and 1,000 mg/dL of protein, respectively. Proteinuria was defined as grades of 1+ or greater.

Other analytical techniques included the hexokinase method for glucose, an enzymatic colorimetric assay for serum lipids, and an immunoradiometric assay for insulin (Biosource, Nivelles, Belgium). Serum uric acid was measured based on the Fossati enzymatic reaction using uricase with a Trinder-like endpoint (ADVIA 1650 Auto Analyzer; Bayer Diagnostics, Leverkusen, Germany). Insulin resistance was assessed with the homeostasis model assessment of insulin resistance (HOMA-IR), according to the following equation: fasting blood insulin (uU/mL)×fasting blood glucose (mmol/L)/22.5. C reactive protein (CRP) was analyzed by particle-enhanced immunonephelometry with the BNII™ System (Dade Behring, Marburg, Germany) using a lower detection limit of 0.175 mg/L. Our clinical laboratory participates annually in the survey by the Korean Association of Quality Assurance for Clinical Laboratories and is accredited by the Korean Laboratory Accreditation Program by the Korean Society for Laboratory Medicine.

Metabolic syndrome was defined according to the modified National Cholesterol Education Program Adult Treatment Panel III [29], as the presence of ≥3 of the following abnormalities: 1) abdominal obesity; 2) fasting glucose ≥100 mg/dL; 3) fasting triglycerides ≥150 mg/dL; 4) HDL cholesterol <40 mg/dL; and 5) blood pressure ≥130/85 mm Hg. Because measurement of waist circumference was not available for all subjects, we substituted abdominal obesity with overall adiposity as a BMI of ≥25 kg/m^2^, which has been proposed as a cut-off for obesity diagnosis of obesity in Asian populations [30].

### Statistical Analyses

One-way ANOVA and χ^2^-tests were used to compare the characteristics of study participants at baseline by quartiles of serum total bilirubin (<0.9, 0.9–1.0, 1.1–1.3, and ≥1.4 mg/dL). This approach was to maintain adequate numbers within each quartile. Participants were followed-up until the development of CKD or the last available visit. The average follow-up period for participants who did not develop CKD was 7.0 years. Since we knew that CKD had occurred between two visits but did not know the precise time of CKD development, we used a parametric Cox model to take into account this type of interval censoring (*stpm* command in Stata) [31]. In these models, the baseline hazard function was parameterized with restricted cubic splines in log time with four degrees of freedom. We estimated adjusted hazard ratios (and 95% confidence intervals) for incident CKD comparing the highest three quartiles of baseline serum bilirubin to the lowest quartile. For time-dependent analyses, we used a pooled logistic regression model that closely approximates a Cox model when the risk of outcome between intervals is low [32].

The models were initially adjusted for age, then for body mass index, smoking, alcohol intake, and exercise, and then further adjusted for ALT, AST, GGT, HOMA-IR, CRP, LDL-cholesterol and metabolic syndrome traits including impaired fasting glucose, high blood pressure, high HDL-cholesterol, high triglycerides and obesity. HOMA-IR and CRP. For testing linear risk trends, we used the quartile rank as a continuous variable in the regression models. We checked the proportional hazards assumption by examining graphs of estimated log(-log) survival.

In sensitivity analysis, we re-examined the association of serum bilirubin with incident proteinuria and with incident CKD defined as an eGFR <64 mL/min/1.73 m^2^, the sex-specific 5^th^ percentile in our study population. Also, to further address potential confounding from liver and hepatobiliary disease, we repeated the analysis excluding participants who had positive serologic markers for hepatitis B or C virus; ultrasound findings of chronic liver disease (including liver cirrhosis, intrahepatic or extrahepatic cholelithiasis, and abnormal dilatation of the biliary tree); or abnormal hepatic function test (total bilirubin ≥2.0 mg/dL, alanine aminotransferase [ALT] ≥80 U/L, aspartate aminotransferase [AST] ≥80 U/L, or gamma-glutamyltransferase [GGT] ≥80 U/L). Statistical analyses were performed using Stata version 11 (StataCorp LP, College Station, TX, USA). All reported P values were 2-tailed, and statistical significance was set at P<0.05.

## Results

At baseline, the mean (SD) age and body mass index of study participants were 37.2 (4.9) years, 24.2 (2.8) kg/m^2^, respectively (Table 1). The median (interquartile range) of serum total, direct and indirect bilirubin levels were 1.10 (0.80–1.40), 0.40 (0.30–0.50) and 0.70 (0.52–0.87) mg/dL, respectively. The prevalences of current smoking, diabetes, hypertension and metabolic syndrome were 46.1, 1.5, 17.2 and 18.5%, respectively. Serum total bilirubin levels were negatively associated with age and with a variety of metabolic parameters, including body mass index, glucose, total cholesterol, LDL-cholesterol, triglycerides, liver enzymes, CRP, insulin and HOMA-IR (Table 1). Serum total bilirubin levels were also negatively associated with current smoking and with the prevalence of the metabolic syndrome while they were positively associated with alcohol intake, regular exercise, uric acid and HDL-cholesterol levels.

**Table 1:** Baseline characteristics of study participants by quartile of serum total bilirubin.

	Overall			Total		bilirubin			
	(N = 12,823)	Quartile 1 (N = 2,847)			Quartile 2 (N = 2,447)		Quartile 3(N = 2,916)	Quartile 4(N = 2,158)	*P* value for trend
Total bilirrubin (mg/dL)†	1.1 (0.80–1.4)		0.7 (0.60–0.8)		1.0 (0.90–1.1)		1.3 (1.20–1.4)	1.7 (1.60–2.0)	
Age (years)*	37.2 (4.9)		37.2 (4.9)		37.4 (5.0)		37.1 (4.8)	36.9 (4.8)	0.04
BMI (kg/m^2^)*	24.2 (2.8)		24.4 (2.8)		24.4 (2.8)		24.0 (2.9)	23.8 (2.8)	<0.001
Current smoker (%)	46.1		53.9		47.3		42.4	38.1	<0.001
Alcohol intake (%)‡	16.3		16.2		15.8		16.6	17.1	0.26
Regular exercise (%)§	50.4		48.7		49.9		50.8	53.1	0.001
Diabetes %	1.5		1.7		1.5		1.2	1.7	0.84
Hypertension %	17.2		16.6		17.5		17.5	17.1	0.60
Metabolic syndrome %	18.5		22.0		19.5		16.6	14.3	<0.001
Systolic BP (mmHg)*	116.1 (13.0)		115.8 (12.7)		116.2 (13.0)		116.0 (13.0)	116.4 (13.4)	0.13
Diastolic BP (mmHg)^*^	75.6 (10.4)		75.2 (10.3)		75.9 (10.5)		75.5 (10.5)	75.7 (10.4)	0.21
Glucose (mg/dL)^*^	92.6 (14.1)		94.1 (14.7)		92.9 (14.0)		91.8 (12.6)	90.9 (14.8)	<0.001
Total cholesterol (mg/dL)^*^	203.1 (35.0)		205.0 (34.4)		205.2 (35.3)		201.8 (34.9)	198.7 (34.8)	<0.001
LDL-C (mg/dL)^*^	120.9 (29.6)		121.3 (29.3)		122.4 (29.9)		120.4 (30.1)	118.5 (28.9)	<0.001
HDL-C (mg/dL)^*^	51.7 (11.4)		50.0 (11.0)		51.2 (11.1)		52.8 (11.6)	53.6 (11.7)	<0.001
Uric acid (mg/dL)^*^	6.08 (1.16)		5.99 (1.20)		6.09 (1.16)		6.13 (1.14)	6.10 (1.14)	<0.001
Creatinine (mg/dL)	1.13 (0.11)		1.13 (0.11)		1.14 (0.11)		1.13 (0.11)	1.13 (0.12)	0.56
GFR (mL/min per 1.73 m^2^)	78.4 (72.0–83.0)		78.8 (72.5–83.2)		78.0 (71.7–82.9)		78.2 (71.8–83.0)	79.0 (72.2–84.4)	0.17
Triglycerides (mg/dL)†	131.0 (94.0–187.0)		141.0 (100.0–205.0)		136.5 (97.0–193.0)		126.0 (91.0–177.0)	120.0 (88.0–169.0)	<0.001
Direct bilirubin (mg/dL)†	0.40 (0.30–0.50)		0.22 (0.20–0.30)		0.36 (0.30–0.40)		0.50 (0.40–0.53)	0.69 (0.60–0.80)	<0.001
Indirect bilirubin (mg/dL)†	0.70 (0.52–0.87)		0.47 (0.40–0.50)		0.61 (0.60–0.70)		0.80 (0.73–0.90)	1.10 (0.98–1.30)	<0.001
ALT (U/l)†	27.0 (19.0–39.0)		28.0 (20.0–41.0)		28.0 (20.0–40.5)		26.0 (19.0–38.0)	25.0 (18.0–37.0)	<0.001
AST (U/l)†	24.0 (20.0–29.0)		24.0 (21.0–29.0)		24.0 (21.0–29.0)		24.0 (20.0–29.0)	24.0 (20.0–29.0)	0.01
GGT (U/l)†	27.0 (18.0–42.0)		28.0 (19.0–44.0)		28.0 (19.0–44.0)		26.0 (18.0–41.0)	24.0 (17.0–39.0)	<0.001
hsCRP (mg/l)†	0.50 (0.30–1.10)		0.60 (0.30–1.30)		0.60 (0.30–1.10)		0.50 (0.30–0.90)	0.40 (0.20–0.80)	<0.001
Insulin (uU/dL)	7.31 (5.50–9.47)		8.07 (6.12–10.12)		7.52 (5.74–9.68)		6.89 (5.36–9.40)	6.62 (5.20–9.03)	<0.001
HOMA-IR†	1.64 (1.23–2.19)		1.83 (1.35–2.35)		1.69 (1.25–2.21)		1.54 (1.17–2.08)	1.48 (1.12–1.97)	<0.001

Abbreviations: ALT, alanine aminotransferase; AST, aspartate aminotransferase; BMI, body mass index; BP, blood pressure; GGT, gamma-glutamyltranspeptidase; HDL-C, high-density lipoprotein-cholesterol; hsCRP, high sensitivity C-reactive protein; HOMA-IR, homeostasis model assessment of insulin resistance; LDL-C: low-density lipoprotein-cholesterol.

Data are *means (standard deviation),

† medians (interquartile range), or percentages.

‡ ≥20 g of ethanol per day.

§ ≥1 time/week.

**Figure 1:**
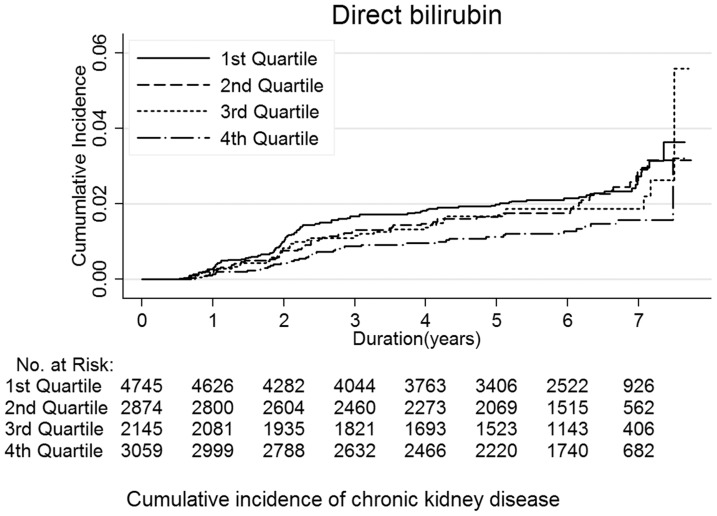
Cumulative incidence of chronic kidney disease by quartile of serum direct bilirubin concentration.

During 70,515.8 person-years of follow-up, 238 participants developed CKD (rate 3.3 per 1,000 person-years). Increasing levels of serum direct bilirubin were progressively associated with decreasing incidence of CKD (Figure 1 and Table 2). In age-adjusted models, the hazard ratios for CKD comparing quartiles 2–4 vs. quartile 1 of serum direct bilirubin were 0.93 (95% confidence interval 0.67–1.28), 0.88 (0.60–1.27) and 0.60 (0.42–0.88), respectively. After adjusting for smoking, alcohol intake and physical activity, the hazard ratio for CKD in the highest quartile compared to the lowest was 0.60 (0.41–0.87; P trend = 0.01). The association persisted after further adjusting for eGFR, liver enzymes and metabolic syndrome traits (hazard ratio comparing the highest to the lowest quartile 0.66 [95% CI 0.45–0.98; P trend = 0.02]) and after introducing serum direct bilirubin and metabolic risk factors as time-dependent exposures (hazard ratio comparing the highest to the lowest quartile 0.43 [95% CI 0.20–0.93; P trend = 0.02]). Further adjustment for hypertension, diabetes, CRP, HOMA-IR or LDL-cholesterol did not materially alter the estimates (data not shown).

**Table 2:** Hazard ratios (95% confidence intervals) for incident chronic kidney disease by serum bilirubin quartiles.

	Person-years	No. of incident cases	Age-adjusted HR(95% CI)	Multivariate HR (95% CI)		Multivariate HR (95% CI), time-dependent model*
				Model 1	Model 2	
Total bilirubin						
<0.9 mg/dl	18,018.4	59	1.00 (reference)	1.00 (reference)	1.00 (reference)	1.00 (reference)
0.9–1.1 mg/dl	23,078.0	820	1.02 (0.73–1.43)	1.01 (0.72–1.41)	1.05 (0.73–1.49)	1.29 (0.82–2.04)
1.2–1.4 mg/dl	15,405.5	56	1.12 (0.78–1.61)	1.11 (0.77–1.60)	1.11 (0.74–1.65)	1.00 (0.62–1.60)
≥1.5 mg/dl	14,013.8	41	0.92 (0.62–1.38)	0.91 (0.61–1.36)	1.03 (0.67–1.59)	0.90 (0.51–1.58)
P for trend			0.89	0.84	0.79	0.56
Direct bilirubin						
<0.31 mg/dl	25,992.4	103	1.00 (reference)	1.00 (reference)	1.00 (reference)	1.00 (reference)
0.31–0.40 mg/dl	15,787.3	58	0.93 (0.67–1.28)	0.93 (0.67–1.29)	0.93 (0.66–1.30)	0.85 (0.56–1.28)
0.41–0.50 mg/dl	11,687.4	38	0.88 (0.60–1.27)	0.88 (0.60–1.28)	0.66 (0.43–1.01)	0.59 (0.33–1.03)
≥0.51 mg/dl	17,048.8	39	0.60 (0.42–0.88)	0.60 (0.41–0.87)	0.66 (0.45–0.98)	0.43 (0.20–0.93)
P for trend			0.01	0.01	0.02	0.02
Indirect bilirubin						
<0.53 mg/dl	17,653.7	53	1.00 (reference)	1.00 (reference)	1.00 (reference)	1.00 (reference)
0.53–0.7 mg/dl	23,370.5	76	1.03 (0.73–1.46)	1.03 (0.72–1.46)	0.94 (0.65–1.36)	1.00 (0.61–1.63)
0.71–0.87 mg/dl	11,851.3	47	1.29 (0.87–1.91)	1.26 (0.85–1.87)	1.07 (0.71–1.62)	1.07 (0.65–1.75)
≥0.88 mg/dl	17,640.3	62	1.21 (0.84–1.75)	1.20 (0.83–1.74)	1.12 (0.76–1.66)	0.80 (0.47–1.36)
P for trend			0.18	0.17	0.44	0.46

Model 1: Adjusted for age, smoking, alcohol intake and exercise.

Model 2: Further adjusted for eGFR, CRP, AST, ALT, GGT and metabolic syndrome traits (high glucose, high blood pressure, low HDL cholesterol, high triglycerides and high BMI).

^*^estimated from pooled logistic regression models with serum bilirubin quartiles as time-dependent categorical variable s adjusted for other covariates (age, smoking, alcohol intake, exercise, eGFR, CRP, AST, ALT, GGT and each metabolic syndrome trait) over time as time-dependent variables.

Abbreviations: CI, confidence interval; eGFR: estimated glomerular filtration rate; HDL, high-density lipoprotein; HR, hazard ratio; LDL, low-density lipoprotein; ALT: alanine aminotransferase; AST: aspartate aminotransferase; GGT: gamma-glutamyltransferase.

The association between serum direct bilirubin and incident CKD was similar across subgroups of study participants, with no significant interactions according to age group (<40 vs ≥40 years of age), BMI group (<25 vs. ≥25 kg/m^2^), metabolic syndrome (presence vs. absence), or lifestyle characteristics (current smoker vs noncurrent smoker, <20 vs ≥20 g of alcohol per day, and <1 time/week vs ≥1 time/week of regular exercise) (data not shown). The results were also similar in the subgroup of participants with no evidence of liver disease at baseline (Table 3).

**Table 3:** Hazard ratios (95% confidence intervals) for incident chronic kidney disease by serum bilirubin quartiles among subjects who did not have any evidence of liver disease (N = 10,348).

	Person-years	No. of incident cases	Age-adjusted HR(95% CI)	Multivariate HR (95% CI)		Multivariate HR (95% CI), time-dependent model^*^
				Model 1	Model 2	
Total bilirubin						
<0.9 mg/dl	15,595.1	51	1.00 (reference)	1.00 (reference)	1.00 (reference)	1.00 (reference)
0.9–1.1 mg/dl	13,384.1	50	1.09 (0.74–1.61)	1.01 (0.72–1.41)	1.05 (0.73–1.49)	1.33 (0.84–2.09)
1.2–1.4 mg/dl	15,928.8	57	1.10 (0.75–1.61)	1.11 (0.77–1.60)	1.11 (0.74–1.65)	1.04 (0.65–1.64)
≥1.5 mg/dl	11,990.8	34	0.91 (0.59–1.40)	0.91 (0.61–1.36)	1.03 (0.67–1.59)	0.90 (0.51–1.58)
P for trend			0.78	0.84	0.79	0.59
Direct bilirubin						
<0.31 mg/dl	22,140.1	91	1.00 (reference)	1.00 (reference)	1.00 (reference)	1.00 (reference)
0.31–0.40 mg/dl	13,546.3	50	0.90 (0.64–1.28)	0.93 (0.67–1.29)	0.93 (0.66–1.30)	0.84 (0.55–1.27)
0.41–0.50 mg/dl	9.877.8	30	0.78 (0.52–1.18)	0.88 (0.60–1.28)	0.66 (0.43–1.01)	0.62 (0.36–1.08)
≥0.51 mg/dl	11,334.5	22	0.50 (0.31–0.80)	0.60 (0.41–0.87)	0.66 (0.45–0.98)	0.43 (0.20–0.92)
P for trend			0.003	0.01	0.02	0.01
Indirect bilirubin						
<0.53 mg/dl	14,420.9	46	1.00 (reference)	1.00 (reference)	1.00 (reference)	1.00 (reference)
0.53–0.7 mg/dl	14,072.5	42	0.88 (0.58–1.34)	1.03 (0.72–1.46)	0.94 (0.65–1.36)	1.01 (0.62–1.64)
0.71–0.87 mg/dl	15,104.0	60	1.21 (0.82–1.77)	1.26 (0.85–1.87)	1.07 (0.71–1.62)	1.10 (0.67–1.80)
≥0.88 mg/dl	13,301.1	45	1.11 (0.73–1.67)	1.20 (0.83–1.74)	1.12 (0.76–1.66)	0.83 (0.49–1.41)
P for trend			0.33	0.17	0.44	0.56

Model 1: Adjusted for age, smoking, alcohol intake and exercise.

Model 2: Further adjusted for eGFR, CRP, AST, ALT, GGT and metabolic syndrome traits (high glucose, high blood pressure, low HDL cholesterol, high triglycerides and high BMI). ^*^estimated from pooled logistic regression models with serum bilirubin quartiles as time-dependent categorical variable s adjusted for other covariates (age, smoking, alcohol intake, exercise, eGFR, CRP, AST, ALT, GGT and each metabolic syndrome trait) over time as time-dependent variables.

Abbreviations: CI, confidence interval; eGFR: estimated glomerular filtration rate; HDL, high-density lipoprotein; HR, hazard ratio; LDL, low-density lipoprotein; ALT: alanine aminotransferase; AST: aspartate aminotransferase; GGT: gamma-glutamyltransferase.

Since eGFR evaluated with a creatinine-dependent formula in subjects with lower BMI may overestimate renal function, we repeated the analyses after excluding participants with BMI of <18.5 (N = 198), but the results were unchanged (data not shown). In addition, we further excluded participants taking drugs that can influence serum bilirubin levels such as barbiturates, tetracyclines, sulfonamides, acetaminophen, benzodiazepines and salicylates (N = 190), but the results did not change (data not shown).

Serum total and indirect bilirubin levels were not significantly related to the incidence of CKD (Table 2). In sensitivity analysis, using an eGFR of <64 mL/min per 1.73 m^2^ to define CKD (Table S1) did not materially alter the estimates. Neither total nor direct or indirect bilirubin concentrations were significantly associated with the incidence of proteinuria (Table S2).

## Discussion

In this longitudinal study of Korean men apparently free of CKD at baseline, higher serum direct bilirubin levels were significantly associated with a lower risk of developing CKD even adjusting for a variety of renal risk factors and cardiometabolic parameters. The association was strong and remained even after adjustment for hypertension and diabetes. Neither serum total bilirubin nor serum indirect bilirubin was significantly associated with CKD incidence.

Our findings are in contrast to the results of Targer et al both in hospital-based unselected outpatients and in the US National Health and Nutrition Examination Survey (NHANES) [24], [25]. There are several potential reasons that can explain this discrepancy. First, the studies of Targer et al. were cross-sectional while our study was longitudinal. The prospective design of our study allows us to establish a clear temporal relationship between serum direct bilirubin levels and incidence of CKD, even after adjusting for multiple cardiometabolic factors, and avoids the problem of reverse causation bias. Second, our study population was of Korean ethnicity, while the representation of Asians in the studies of Targer et al. was limited. We also note that the findings of other studies in Asian populations are consistent with our findings [20]–[22]. Third, in addition to differences in ethnicity, our population is also very different from hospital-based and from NHANES populations in age distribution, anthropometry, comorbidities, and medication use, all factors that may affect bilirubin levels and potentially the association between bilirubin and incident CKD. Finally, we note that in our study all participants had consistent fasting times, while in NHANES only a fraction of participants had fasting samples, and even fasting participants had varying fasting times, which can markedly affect bilirubin levels. Our study thus extends previous findings using prospective data from a large cohort study, thus establishing without ambiguity that changes in bilirubin levels precede the development of CKD.

Bilirubin is formed by breakdown of heme [5]. Heme ring-opening by heme oxygenase, results in the formation of biliverdin, which is reduced to bilirubin by biliverdin reductase [33]. Unconjugated bilirubin is extremely poorly soluble in water, and it is strongly bound to albumin in plasma [34]. In hepatocytes, bilirubin is conjugated with one or two molecules of glucuronic acid to bilirubin monoglucuronide or diglucuronide (conjugated or direct bilirubin), a reaction catalyzed by bilirubin-UDP-glucuronosyltransferase [35]. In comparion to unconjugated bilirubin, conjugated bilirubin is soluble in plasma and only weakly bound to albumin, and thus more easily available in active form compared to indirect bilirubin [36]. Direct bilirubin is also easily excreted in the bile and can be filtered by the kidney [34]. While our study found that the negative association between bilirubin and CKD incidence was restricted to direct bilirubin, most previous studies of bilirubin levels and cardiometabolic endpoints measured only total bilirubin without separating bilirubin types. There is very limited data showing a differentially protective effect of direct bilirubin on metabolic syndrome and its components [14], [37]. Further studies on the role of each type of serum bilirubin on the development of CKD are clearly needed.

The mechanisms by which serum direct bilirubin levels are associated with a lower risk of CKD need to be elucidated. Systemic low-grade inflammation as assessed by CRP could represent a possible mechanism linking serum bilirubin with CKD development. Several studies have reported that serum bilirubin was negatively correlated with CRP [38], [39]. A previous study showed that low grade inflammation may be a predictor for a change in kidney function in the elderly [40], but the association between serum direct bilirubin levels and CKD persisted after adjusting for CRP in our study. Insulin resistance may also explain the serum bilirubin-incident CKD relationship, because serum bilirubin levels have been negatively associated with insulin resistance and with the metabolic syndrome [14], [41] and several studies reported that insulin resistance was associated with an increased risk for CKD [42]. In our study, however, the risk for CKD decreased with increasing quartiles of serum direct bilirubin independently of HOMA-IR and metabolic syndrome.

The association of the serum bilirubin with the incidence of CKD could also be explained by oxidative stress. In our study, serum bilirubin concentrations were negatively associated with serum GGT which has been proposed as a sensitive and reliable marker of oxidative stress [43]. Oxidative stress and renal reactive oxygen species induce renal vasoconstriction, sodium retention, and kidney damage [44], [45]. Bilirubin may have strong antioxidant properties [5], [46]. Rat kidneys flushed with 10 µmol/l bilirubin demonstrated significant improvements in urine output, GFR, tubular function, and mitochondrial integrity after 20 min of warm ischemia [8] and superinduction of heme oxygenase lead to bilirubin-mediated reductions in oxidative stress following renal ischemia [7], [47]. In a rat model, bile duct ligation-induced cholestasis showed protective effect against renal injury [48].

Serum bilirubin concentrations were higher in our population compared to other Western general populations. Several factors may be responsible for these high levels. First, it is well known that serum bilirubin concentrations are significantly higher in men than in women, and they tend to decline with advancing age in both genders [49]. Our study population was comprised only of young Korean men (mean [SD] age, 37.1 [4.9] years), which may result in higher average levels compared to a general, unselected population. Second, there may be ethnic differences in bilirubin levels. Black adults, for instance, have lower bilirubin concentration than White adults [50], [51], while Asian infants have higher bilirubin concentrations than White infants [52]–[54]. Mean maximal serum concentrations of unconjugated bilirubin in full-term Japanese, Korean, Chinese and American Indian newborns are approximately double than those found in White and Black populations [55]. While we have not found any papers comparing bilirubin levels in Asian vs. Caucasian adult populations, we note that other studies in different populations in Korea also report bilirubin levels comparable to ours or even higher [37]. Bilirubin data is not available in Korean NHANES, so we cannot compare our result with that of Korean NHANES. Other reports using Korean population (about 50 s) also showed that the mean serum total bilirubin levels were 1.02 (0.80–1.30) [14]. Finally, it is also possible that the long fasting times in our study (>12 h) might be related to higher bilirubin concentration, as longer fasting times have been related to higher bilirubin concentrations [56], [57].

Several limitations also need to be considered in the interpretation of our findings. We used estimated GFR instead of a directly measured GFR to define CKD, adding substantial random variability to our results. In addition, GFR estimating equations are not well-validated in Asian populations. These sources of error, however, are non-differential with respect to bilirubin levels and if anything will result in underestimation of the negative association between serum direct bilirubin and incident CKD. Also, subjects in this study were male Korean workers with an average body mass index of 24 kg/m^2^ attending health screening exams regularly. This population is likely to be healthier than the general population. As a consequence, our findings may not be generalizable to females, to populations with larger body mass, or to non-working populations. Despite these potential limitations, this study is the first prospective study to demonstrate a negative association between serum direct bilirubin and incident CKD. Strengths of our study include the large sample size, its prospective design, the availability of detailed health exam and laboratory information, and the relative homogeneity of its participants, which reduces confounding by such variables as socioeconomic status and access to health care.

## Conclusions

Higher serum direct bilirubin levels were significantly associated with a lower risk of the development of CKD even adjusting for a variety of cardiometabolic parameters. Thus, serum direct bilirubin may become a new marker for assessing CKD risk and a potential target for risk modification. Further studies are needed, however, to elucidate the mechanisms underlying the negative association between serum direct bilirubin and CKD.

## Supporting Information

Table S1 **Association of serum bilirubin with incident chronic kidney disease (CKD) after defining incident CKD as eGFR <64 mL/min/1.73 m^2^ (5^th^ percentile in our study population).**
(DOC)

Table S2 **Association of serum bilirubin with incident proteinuria.**
(DOC)
