# Supplementary material for: Higher Serum Direct Bilirubin Levels Were Associated with a Lower Risk of Incident Chronic Kidney Disease in Middle Aged Korean Men
Source: PLoS One. 2014 Feb 20;9(2):e75178. doi: 10.1371/journal.pone.0075178 (PMC3930500; doi:10.1371/journal.pone.0075178)
Supplement: Table S1 — Association of serum bilirubin with incident chronic kidney disease (CKD) after defining incident CKD as eGFR <64 mL/min/1.73 m2 (5th percentile in our study population). (DOC) [file pone.0075178.s001.doc]

**Table S1.** Association of serum bilirubin with incident chronic kidney disease (CKD) after defining incident CKD as eGFR <64 mL/min/1.73m^2^ (5^th^ percentile in our study population).

|  | Person-years | No. of incident  cases | Age-adjusted HR  (95% CI) | Multivariate  (95% | HR  CI) | Multivariate HR  (95% CI), |
| --- | --- | --- | --- | --- | --- | --- |
|  |  |  |  | Model 1 | Model 2 | time-dependent  model^*^ |
| Total bilirubin |  |  |  |  |  |  |
| <0.9 mg/dl | 17,489.1 | 150 | 1.00 (reference) | 1.00 (reference) | 1.00 (reference) | 1.00 (reference) |
| 0.9 – 1.1 mg/dl | 22,348.9 | 213 | 1.05 (0.85 – 1.29) | 1.03 (0.84 – 1.28) | 1.02 (0.82 – 1.28) | 1.30 (0.80 – 2.11) |
| 1.2 – 1.4 mg/dl | 14,986.4 | 128 | 1.00 (0.79 – 1.27) | 0.98 (0.77 – 1.24) | 1.01 (0.78 – 1.30) | 1.09 (0.67 – 1.79) |
| ≥1.5 mg/dl | 13,654.2 | 84 | 0.73 (0.56 – 0.96) | 0.71 (0.54 – 0.93) | 0.78 (0.59 – 1.04) | 0.92 (0.52 – 1.64) |
| P for trend |  |  | 0.03 | 0.02 | 0.14 | 0.70 |
| Direct bilirubin |  |  |  |  |  |  |
| <0.31 mg/dl | 25,164.7 | 255 | 1.00 (reference) | 1.00 (reference) | 1.00 (reference) | 1.00 (reference) |
| 0.31 – 0.40 mg/dl | 15,324.7 | 145 | 0.94 (0.77 – 1.15) | 0.93 (0.76 – 1.14) | 0.90 (0.73 – 1.12) | 0.97 (0.64 – 1.48) |
| 0.41 – 0.50 mg/dl | 11,370.7 | 83 | 0.76 (0.59 – 0.97) | 0.75 (0.59 – 0.97) | 0.62 (0.47 – 0.81) | 0.58 (0.32 – 1.04) |
| ≥0.51 mg/dl | 16,618.5 | 92 | 0.57 (0.45 – 0.72) | 0.55 (0.43 – 0.70) | 0.61 (0.48 – 0.79) | 0.53 (0.26 – 1.05) |
| P for trend |  |  | <0.001 | <0.001 | <0.001 | 0.02 |
| Indirect bilirubin |  |  |  |  |  |  |
| <0.53 mg/dl | 17,182.6 | 134 | 1.00 (reference) | 1.00 (reference) | 1.00 (reference) | 1.00 (reference) |
| 0.53 – 0.7 mg/dl | 22,694.3 | 199 | 1.07 (0.86 – 1.33) | 1.06 (0.85 – 1.32) | 0.97 (0.77 – 1.22) | 1.10 (0.65 – 1.85) |
| 0.71 – 0.87 mg/dl | 11,453.9 | 113 | 1.24 (0.97 – 1.59) | 1.21 (0.94 – 1.56) | 1.07 (0.83 – 1.39) | 1.31 (0.77 – 2.23) |
| ≥0.88 mg/dl | 17,147.8 | 129 | 1.00 (0.78 – 1.27) | 0.97 (0.76 – 1.24) | 0.95 (0.73 – 1.23) | 0.90 (0.52 – 1.57) |
| P for trend |  |  | 0.78 | 0.82 | 0.92 | 0.80 |

Model 1: Adjusted for age, smoking, alcohol intake and exercise.

Model 2: Further adjusted for eGFR, AST, ALT, GGT and metabolic syndrome traits (high glucose, high blood pressure, low HDL cholesterol, high triglycerides and high BMI).

^*^estimated from pooled logistic regression models with serum bilirubin quartiles as time-dependent categorical variable s adjusted for other covariates (age, smoking, alcohol intake, exercise, eGFR, AST, ALT, GGT and each metabolic syndrome trait) over time as time-dependent variables.

Abbreviations: CI, confidence interval; eGFR: estimated glomerular filtration rate; HDL, high-density lipoprotein; HR, hazard ratio; LDL, low-density lipoprotein; ALT: alanine aminotransferase; AST: aspartate aminotransferase; GGT: gamma-glutamyltransferase
