# Supplementary material for: Higher Serum Direct Bilirubin Levels Were Associated with a Lower Risk of Incident Chronic Kidney Disease in Middle Aged Korean Men
Source: PLoS One. 2014 Feb 20;9(2):e75178. doi: 10.1371/journal.pone.0075178 (PMC3930500; doi:10.1371/journal.pone.0075178)
Supplement: Table S2 — Association of serum bilirubin with incident proteinuria. (DOC) [file pone.0075178.s002.doc]

**Table S2.** Association of serum bilirubin with incident proteinuria.

|  | Person-years | No. of incident  cases | Age-adjusted HR  (95% CI) | Multivariate  (95% | HR  CI) | Multivariate HR  (95% CI), |
| --- | --- | --- | --- | --- | --- | --- |
|  |  |  |  | Model 1 | Model 2 | time-dependent  model^*^ |
| Total bilirubin |  |  |  |  |  |  |
| <0.9 mg/dl | 18,103.3 | 189 | 1.00 (reference) | 1.00 (reference) | 1.00 (reference) | 1.00 (reference) |
| 0.9 – 1.1 mg/dl | 23,162.5 | 243 | 1.03 (0.86 – 1.24) | 1.06 (0.88 – 1.27) | 1.11 (0.92 – 1.34) | 1.11 (0.75 – 1.66) |
| 1.2 – 1.4 mg/dl | 15,405.6 | 143 | 0.92 (0.75 – 1.13) | 0.95 (0.78 – 1.17) | 1.03 (0.83 – 1.27) | 0.93 (0.62 – 1.40) |
| ≥1.5 mg/dl | 14,068.6 | 140 | 0.95 (0.77 – 1.18) | 1.00 (0.81 – 1.24) | 1.05 (0.84 – 1.32) | 0.95 (0.61 – 1.49) |
| P for trend |  |  | 0.42 | 0.75 | 0.83 | 0.65 |
| Direct bilirubin |  |  |  |  |  |  |
| <0.31 mg/dl | 26,084.4 | 298 | 1.00 (reference) | 1.00 (reference) | 1.00 (reference) | 1.00 (reference) |
| 0.31 – 0.40 mg/dl | 15,897.9 | 142 | 0.76 (0.63 – 0.92) | 0.78 (0.64 – 0.94) | 0.83 (0.69 – 1.02) | 0.93 (0.65 – 1.33) |
| 0.41 – 0.50 mg/dl | 11,663.8 | 125 | 0.95 (0.78 – 1.15) | 0.97 (0.79 – 1.18) | 1.04 (0.85 – 1.28) | 0.62 (0.38 – 1.02) |
| ≥0.51 mg/dl | 17,093.8 | 150 | 0.74 (0.62 – 0.90) | 0.77 (0.64 – 0.93) | 0.85 (0.69 – 1.04) | 0.69 (0.42 – 1.13) |
| P for trend |  |  | 0.01 | 0.02 | 0.26 | 0.05 |
| Indirect bilirubin |  |  |  |  |  |  |
| <0.53 mg/dl | 17,701.3 | 175 | 1.00 (reference) | 1.00 (reference) | 1.00 (reference) | 1.00 (reference) |
| 0.53 – 0.7 mg/dl | 23,466.1 | 243 | 1.12 (0.93 – 1.34) | 1.13 (0.94 – 1.36) | 1.14 (0.94 – 1.38) | 1.03 (0.68 – 1.57) |
| 0.71 – 0.87 mg/dl | 11,913.3 | 118 | 1.05 (0.84 – 1.31) | 1.09 (0.87 – 1.36) | 1.13 (0.90 – 1.43) | 0.98 (0.63 – 0.52) |
| ≥0.88 mg/dl | 17,659.3 | 179 | 1.02 (0.84 – 1.25) | 1.07 (0.87 – 1.31) | 1.08 (0.87 – 1.33) | 0.85 (0.55 – 1.31) |
| P for trend |  |  | 0.97 | 0.57 | 0.57 | 0.39 |

Model 1: Adjusted for age, smoking, alcohol intake and exercise.

Model 2: Further adjusted for eGFR, AST, ALT, GGT and metabolic syndrome traits (high glucose, high blood pressure, low HDL cholesterol, high triglycerides and high BMI).

^*^estimated from pooled logistic regression models with serum bilirubin quartiles as time-dependent categorical variable s adjusted for other covariates (age, smoking, alcohol intake, exercise, eGFR, AST, ALT, GGT and each metabolic syndrome trait) over time as time-dependent variables.

Abbreviations: CI, confidence interval; eGFR: estimated glomerular filtration rate; HDL, high-density lipoprotein; HR, hazard ratio; LDL, low-density lipoprotein; ALT: alanine aminotransferase; AST: aspartate aminotransferase; GGT: gamma-glutamyltransferase
